# The Role of Verapamil Toxicity in the Vicious Cycle of Bradycardia, Renal Failure, Atrioventricular Nodal Blockade, Shock, and Hyperkalemia (BRASH) Syndrome: A Case Report

**DOI:** 10.7759/cureus.32336

**Published:** 2022-12-08

**Authors:** Yasmin H Kazim, Farnoosh J Farzin, Ivyan Kambal, Omar Q Muhammed Nouri

**Affiliations:** 1 Emergency Department, Rashid Hospital/Dubai Health Authority, Dubai, ARE; 2 Emergency Department, Dubai Health Authority, Dubai, ARE

**Keywords:** verapamil mechanism of action, acute kidney injury and brash syndrome, pathophysiology of brash syndrome, brash syndrome, verapamil toxicity

## Abstract

BRASH is an acronym describing the vicious cycle seen in patients taking atrioventricular (AV) nodal blockers who tend to present with bradycardia, renal failure, atrioventricular nodal blockade, shock, and hyperkalemia. Herein, we report the case of an 87-year-old hypertensive patient on verapamil who presented with complaints of fever and shortness of breath. She was found to have bradycardia, hyperkalemia, renal impairment, and borderline hypotension. Differentiating this case from previous case reports on BRASH syndrome, this patient was found to simultaneously have toxic levels of serum verapamil.

## Introduction

The BRASH syndrome is a collective of bradycardia, renal failure, atrioventricular nodal blockade, shock, and hyperkalemia recently surfacing in literature [1]. Frequently, this syndrome presents alongside hyperkalemia-associated arrhythmias and has emerged as a separate entity as the typical mild hyperkalemia findings in patients did not correlate with electrocardiographic findings [2-5]. Ultimately, patients with BRASH syndrome progress to have multi-organ failure. Among these reported cases, contributory factors such as renal impairment have been suggested to worsen the effects of mildly elevated potassium levels. We present a case of verapamil toxicity as a contributory factor to BRASH syndrome and its contribution to the vicious cycle of bradycardia, worsening renal failure, increasing hyperkalemia, and, in turn, increasing serum verapamil levels.

## Case presentation

An 87-year-old female, known to have type 2 diabetes mellitus, hypertension, and dyslipidemia, presented to the emergency department (ED) with complaints of fever, shortness of breath, chest pain, and fatigue for one day. Her home medications included sustained-release (SR) verapamil 240 mg, gliclazide 30 mg daily, and atorvastatin 40 mg daily. There was no history of a possible overdose of medication

**Figure 1:**
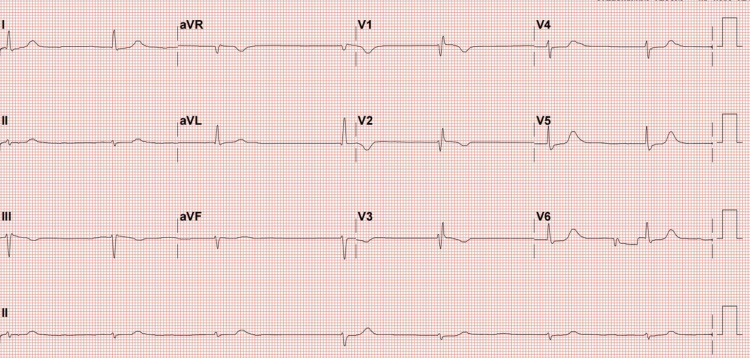
The patient’s ECG on presentation showing a sinus arrest with an escape junctional rhythm and an incomplete right bundle branch block ECG: electrocardiography

**Figure 2:**
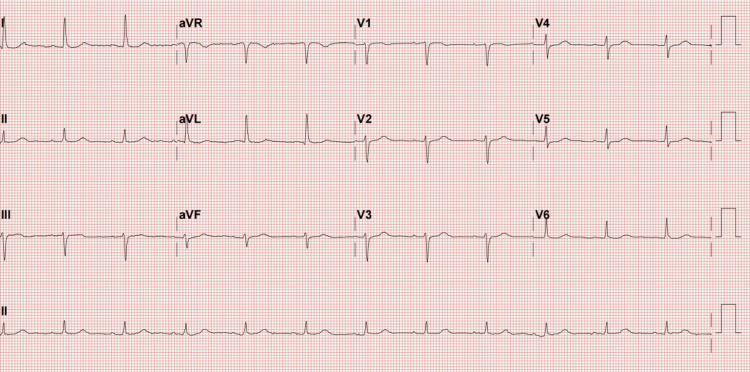
The patient’s ECG four months prior to presentation indicating a normal ECG ECG: electrocardiography

On presentation, her vital signs revealed bradycardia with a heart rate of 42 beats per minute, blood pressure of 112/36 mmHg, 78% SpO2, respiratory rate of 24 breaths per minute, and temperature of 36°C. The electrocardiography (ECG) revealed a sinus arrest with an escape junctional rhythm and an incomplete right bundle branch block (Figure 1). These were considered new changes as her previous ECG four months ago was apparently normal (Figure 2).

Upon physical examination, the patient was conscious and oriented. She had decreased air entry in bilateral lung bases. The remaining systemic examination, however, was unremarkable. Positive laboratory findings are summarized in Table 1.

**Table 1:** Positive laboratory findings Normal ranges are provided between brackets. FBC: full blood count, WBC: white blood cell, eGFR: estimated glomerular filtration rate, CKD-EPI: Chronic Kidney Disease Epidemiology Collaboration

FBC	
WBC count	15.5 × 10^3^/uL (3.6-11 × 10^3^/uL)
Neutrophil	83.9%
Lymphocyte	8.3%
C-reactive protein	227.7 mg/L (<5 mg/L)
Procalcitonin	0.82 ng/mL (<0.05 ng/mL)
Creatinine	1.8 mg/dL (0.7-1.2 mg/dL)
eGFR (CKD-EPI)	26.9 mL/minute/1.73 m^2^ (>60 mL/minute/1.73 m^2^)
Urea and electrolytes	
Potassium	5.6 mmol/L (3.3-4.8 mmol/L)
Urea	81 mg/dL (12-40 mg/dL)
Verapamil level	1,670+++ ng/mL (20-250 ng/mL)

**Figure 3:**
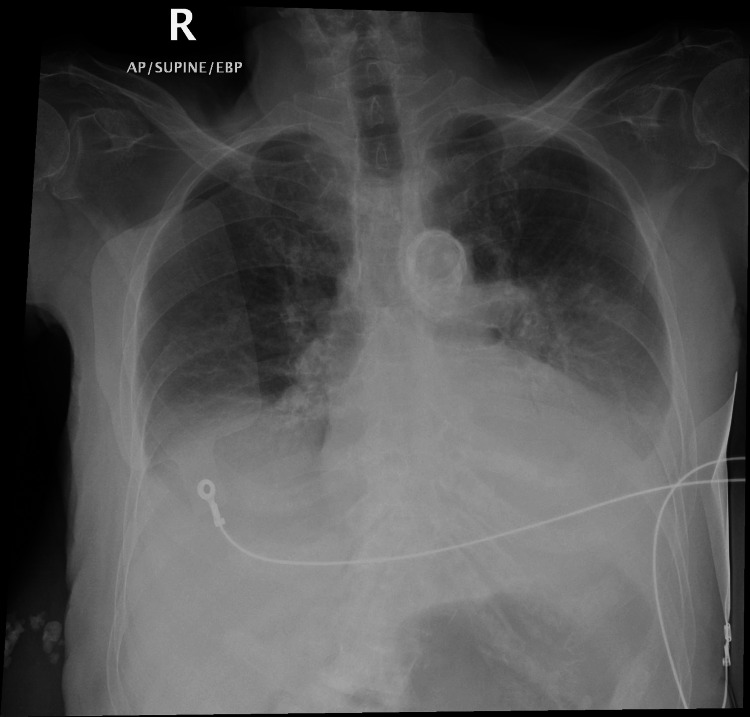
The patient’s chest X-ray indicating pneumonia

These findings were suggestive of an acute kidney injury, hyperkalemia, and an infective process indicated by the elevated inflammatory markers. Serum verapamil level was reported as above laboratory alert level despite being on therapeutic doses. Chest X-ray reportedly showed bilateral pleural effusions with bilateral lower lung zone airspace consolidations signifying pneumonia (Figure 3).

Bedside echocardiography was done, revealing an ejection fraction of 60% with associated bradycardia. During the reassessment of the patient, her heart rate dropped to 30 beats per minute, and 0.6 mg of atropine was administered. Unfortunately, the heart rate failed to improve, and shortly after, she went into cardiac arrest. Cardiopulmonary resuscitation (CPR) was commenced as per the advanced cardiac life support (ACLS) algorithm. The cardiac monitor showed asystole; three cycles of CPR were performed prior to achieving return of spontaneous circulation (ROSC). During the resuscitation, a venous blood gas sample showed hyperkalemia with a potassium level of 6.2 mmol/L. The patient was given 1 g of calcium gluconate IV, insulin, dextrose IV, and a salbutamol nebulizer.

She remained unstable after ROSC with a heart rate ranging from 20 to 30 beats per minute, a palpable central pulse, and an unrecordable blood pressure necessitating the use of transcutaneous pacing along with an epinephrine infusion. Due to hypoventilation and inadequate chest expansion, she was intubated using rapid sequence intubation.

Soon after, her heart rate improved to 60 beats per minute, and her systolic blood pressure picked up to about 110 mmHg. The cardiology team then switched the transcutaneous pacing to transvenous pacing where thereafter the patient remained hemodynamically stable.

## Discussion

**Figure 4:**
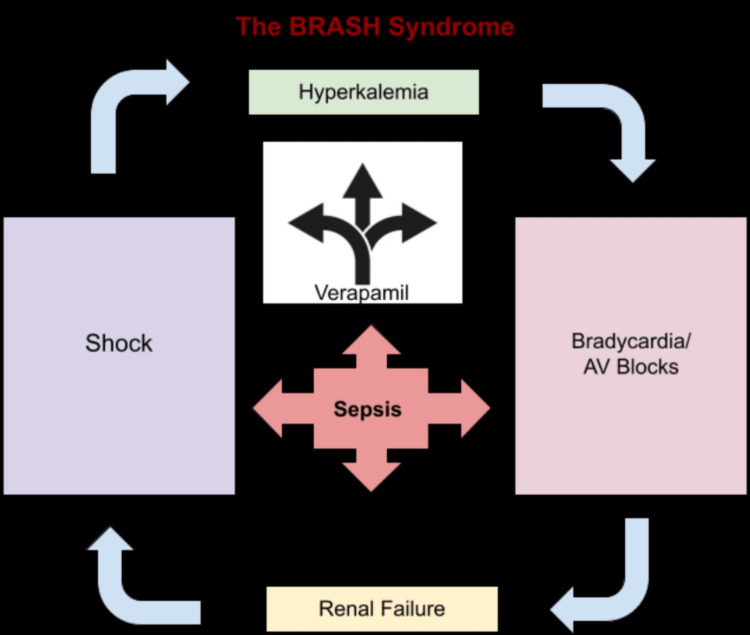
The vicious cycle of BRASH syndrome (based on the findings in the case presented) BRASH: bradycardia, renal failure, atrioventricular nodal blockade, shock, and hyperkalemia

The BRASH syndrome is characterized by the presence of bradycardia, renal failure, atrioventricular nodal blockade medication, shock, and hyperkalemia, whereby each of the named symptoms plays a continuous role in sustaining the vicious cycle of BRASH by magnifying and worsening the effects of each other (Figure 4).

Verapamil falls under the phenylalkylamines (non-dihydropyridine) subgroup of calcium channel blockers (CCB). It acts by inhibiting voltage-dependent L-type calcium channels, causing relaxation of vascular smooth muscle and in turn resulting in a negative inotropic and chronotropic cardiac effect [6]. Verapamil binds to calcium channels at the level of the sinoatrial (SA) and atrioventricular (AV) nodes and results in a negative chronotropic effect [7].

Verapamil is mainly metabolized in the liver, and approximately 70% of its metabolites are excreted through the urine. Hepatic and renal failure may prolong its half-life [8]. While acute administration of verapamil has been shown to result in an increased hepatic and renal blood flow, this is not seen in chronic administration, which can result in delayed clearance of the drug in those on chronic use [9]. The patient’s creatinine level was elevated (1.8 mg/dL), and she had an acute impaired renal function (eGFR: 26.9 mL/minute), which could have contributed to the toxic levels of serum verapamil.

Toxic levels of non-dihydropyridine CCBs can present with ECG findings of sinus bradycardia and various conduction abnormalities including AV blocks, QT prolongation, and heart block. While the patient had no history of underlying cardiac pathology prior to the events of this case, she did have bradycardia coupled with an escape junctional rhythm, which is likely a result of high levels of serum verapamil [10].

The critical pathophysiological characteristic of this syndrome involves a synergistic effect of mild hyperkalemia and therapeutic doses of AV nodal blocker medications resulting in significant bradycardia [2]. Similarly, in our case, the patient had a potassium level of 5.6 mEq/L and was on therapeutic doses of verapamil 240 mg. In the presence of a systemic infection, she appears to have developed acute renal impairment leading to severe and unstable bradycardia rapidly deteriorating into cardiac arrest. In addition, the development of acute renal failure allowed for the accumulation of serum verapamil leading to toxic levels (Figure 4).

## Conclusions

Conclusively, we can say that sepsis contributed to the findings of acute renal failure, metabolic acidosis, and, in turn, the reduced excretion of verapamil. The sepsis was also a potential cause of the hypotension. However, by adding a toxic level of verapamil to the equation, one could also argue that verapamil would have a synergistic effect toward hypotension, bradycardia, metabolic acidosis, and mild hyperkalemia. This could further be supported by the fact that the bradycardia eventually required cardiac pacing to stabilize the patient after her cardiac arrest. Such cases of renal dysfunction, as seen in this case of sepsis, can swiftly potentiate verapamil toxicity. The effects of verapamil toxicity are the same effects gathered under the acronym of the BRASH syndrome.
